# Supplementary material for: Dual functions of microRNA-17 in maintaining cartilage homeostasis and protection against osteoarthritis
Source: Nat Commun. 2022 May 4;13:2447. doi: 10.1038/s41467-022-30119-8 (PMC9068604; doi:10.1038/s41467-022-30119-8)
Supplement: Supplementary file 1 — Supplementary Information [file 41467_2022_30119_MOESM1_ESM.pdf]

SUPPLEMENTARY INFORMATION

Dual functions of microRNA-17 in maintaining cartilage homeostasis and protection against osteoarthritis

Yun Zhang, Shuaijun Li, Peisheng Jin, Ting Shang, Ruizhu Sun, Laiya Lu, Kaijin Guo, Jiping Liu, Yongjuan Tong, Junbang Wang, Sanhong Liu, Chen Wang, Yubin Kang, Wenmin Zhu, Qian Wang, Xiaoren Zhang, Feng Yin, Yi Eve Sun, Lei Cui

Supplementary Fig. 1

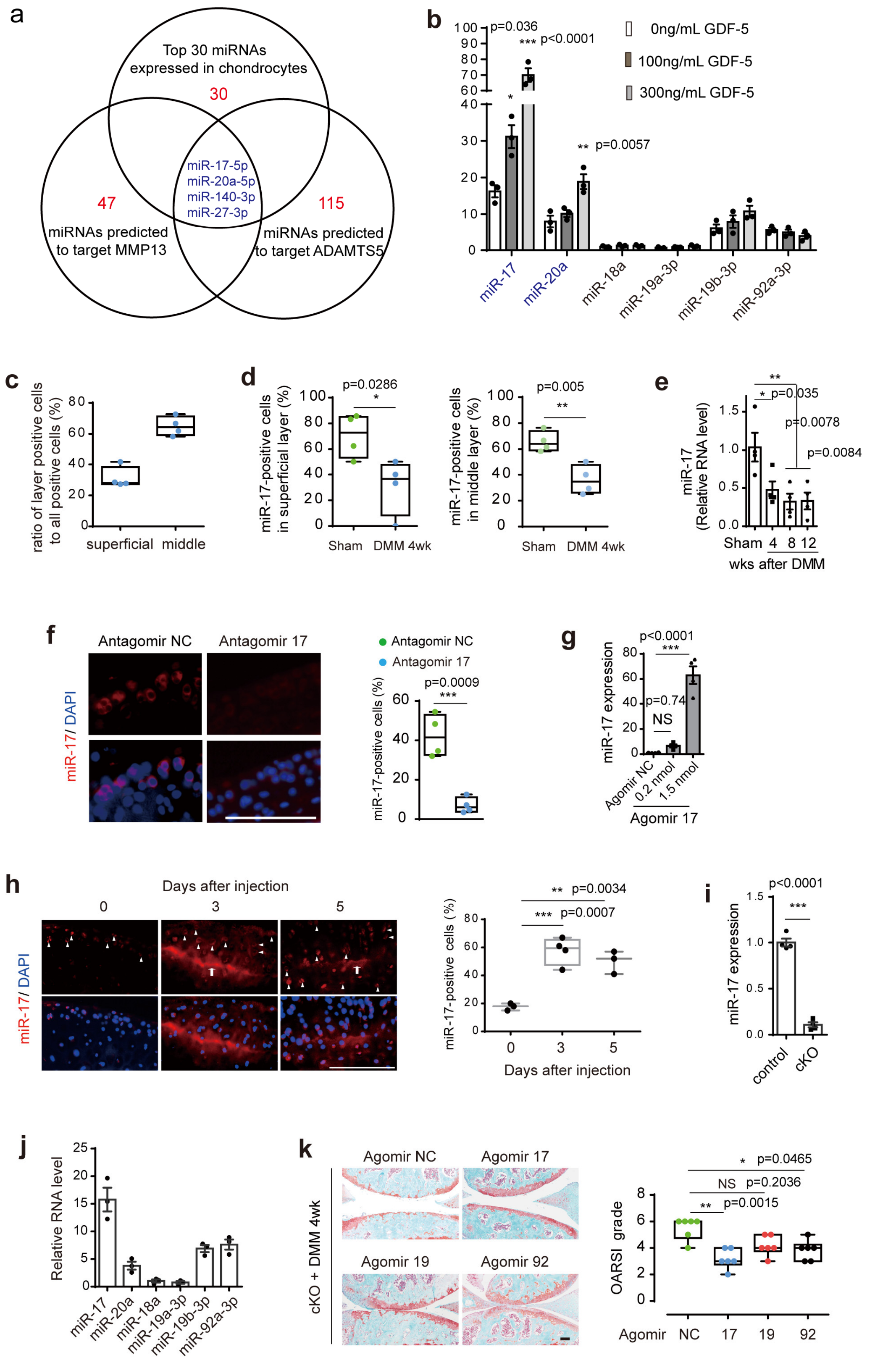

Supplementary Fig. 1 The expression and interference of miR-17 or *miR-17-92* cluster in normal mice and OA models.

(a) In top 30 miRNAs expressed in chondrocytes, four miRNAs were predicted to target MMP13 and ADAMTS5 simultaneously by TargetScan and miRanda. The lists of miRNAs predicted to target MMP13 or ADAMTS5 and the top 30 miRNAs in chondrocytes were provided in source data.

(b) Expression of members of the *miR-17~92* cluster, as determined by qRT-PCR analysis, in primary cultures of mouse articular chondrocytes treated with GDF-5 (0, 100 or 300 ng/mL) for 24 h. n = 3 biologically independent samples per group.

(c) The ratio of miR-17 positive cells in superficial or middle layers to all miR-17 positive cells in healthy cartilage. n = 4 mice.

(d) Percentage of miR-17-positive cells over total DAPI^{+} cells within superficial or middle layer of sham-operated or 4 weeks after DMM mice. n = 4 mice per group.

(e) qRT-PCR analysis of miR-17 expression in joint cartilage from DMM and sham-operated mice. n= 4 biologically independent samples at each timepoint.

(f) FISH analysis of miR-17 and quantification of miR-17-positive cells in mice subjected to intra-articular injection of antagomir-NC (n = 4 mice) or antagomir-17 (n = 4 mice) at 3 days after injections.

(g) qRT-PCR analysis of miR-17 in articular cartilage 3 days after intra-articular injection of agomir-17 (0.2 and 1.5 nmol) or 1.5 nmol of negative control agomir (agomir-NC). The injections were performed 4 weeks after DMM operation. n = 4 biologically independent samples per group.

(h) Representative images from the FISH analysis of miR-17 and quantification of miR-17-positive cells in joint cartilage at the indicated days after agomir-17 (1.5 nmol) injection. The white triangles indicate chondrocytes and arrows indicate accumulation of agomir-17 in matrix. n= 3 mice at day 0 and day 5, n= 4 mice at day 3.

(i) qRT-PCR analysis of miR-17 expression in articular cartilage from *miR-17~92* cKO mice at 1 week after tamoxifen injection. Cre- mice (*miR-17~92*$^{fl/fl}$) were used as controls. n = 4 biologically independent samples per group.

(j) Relative expression level of miRNA members belonging to *miR-17-92* cluster in normal mouse articular cartilage. n = 3 biologically independent samples.

(k) Representative images of safranin O/fast green staining and OARSI grade from *miR-17~92* cKO mice at 4 weeks after DMM surgery. Agomir-NC, agomir-17, agomir-19 or agomir-92 (2 nmol/injection) were injected weekly for 3 times beginning at 1 week after surgery. n= 6 mice per group.

Data are presented as mean ± s.e.m. or boxplots. Box plots: center line, median; box limits, 25th to 75th percentiles; whiskers, min to max. NS, not significant, *$P < 0.05$, **$P < 0.01$, ***$P < 0.001$. Two-sided Student's t-test for d, f, i; one way ANOVA with Bonferroni's test for b, e, g, h; one way ANOVA (nonparametric) with Dunn's multiple comparisons test for k. All scale bars, 100 μm. Source data are provided as a Source Data file.

Supplementary Fig. 2

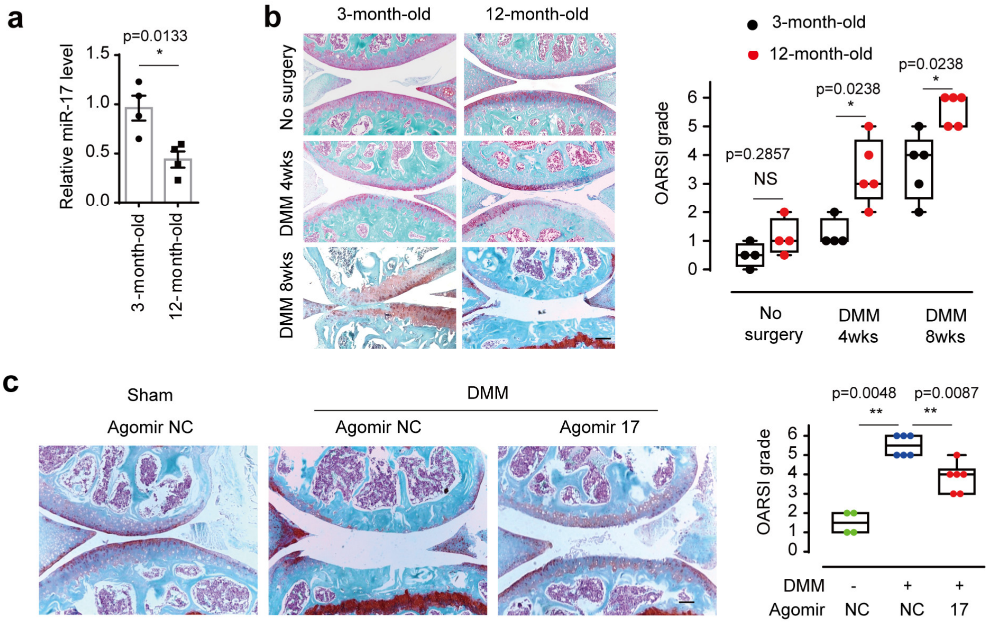

Supplementary Fig. 2 miR-17 supplementation protected against cartilage destruction in DMM cartilage of aged mice.

(a) qRT-PCR analysis of miR-17 expression in knee joint cartilage from 3- or 12-month-old mice. n = 4 biologically independent samples per group.

(b) Safranin O staining of joint sections and OARSI scores from mice at 4 or 8 weeks after DMM surgery. The surgery was performed on mice at the age of 3- or 12-month-old. n = 4 mice (3-month-old no surgery, 12-month-old no surgery, 3-month-old DMM 4wks); n= 5 mice (12-month-old DMM 4wks, 3-month-old DMM 8wks, 12-month-old DMM 8wks).

(c) Representative images of safranin O/fast green staining of joint sections and OARSI scores from 12-month-old mice at 8 weeks after DMM surgery. Agomir-NC or agomir-17 (1.5 nmol) was injected into the knee joints 4 weeks after DMM surgery. n=4 mice (sham+agomir NC); n=6 mice (DMM+agomir NC, DMM+agomir 17).

Data are presented as mean ± s.e.m. or boxplots. Box plots: center line, median; box limits, 25th to 75th percentiles; whiskers, min to max. NS, not significant, **P*< 0.05, ***P*< 0.01, ****P*< 0.01. Two-sided Student's t-test for a; two-sided Mann-Whitney *U* test for OARSI grade. All scale bars, 100 μm. Source data are provided as a Source Data file.

Supplementary Fig. 3

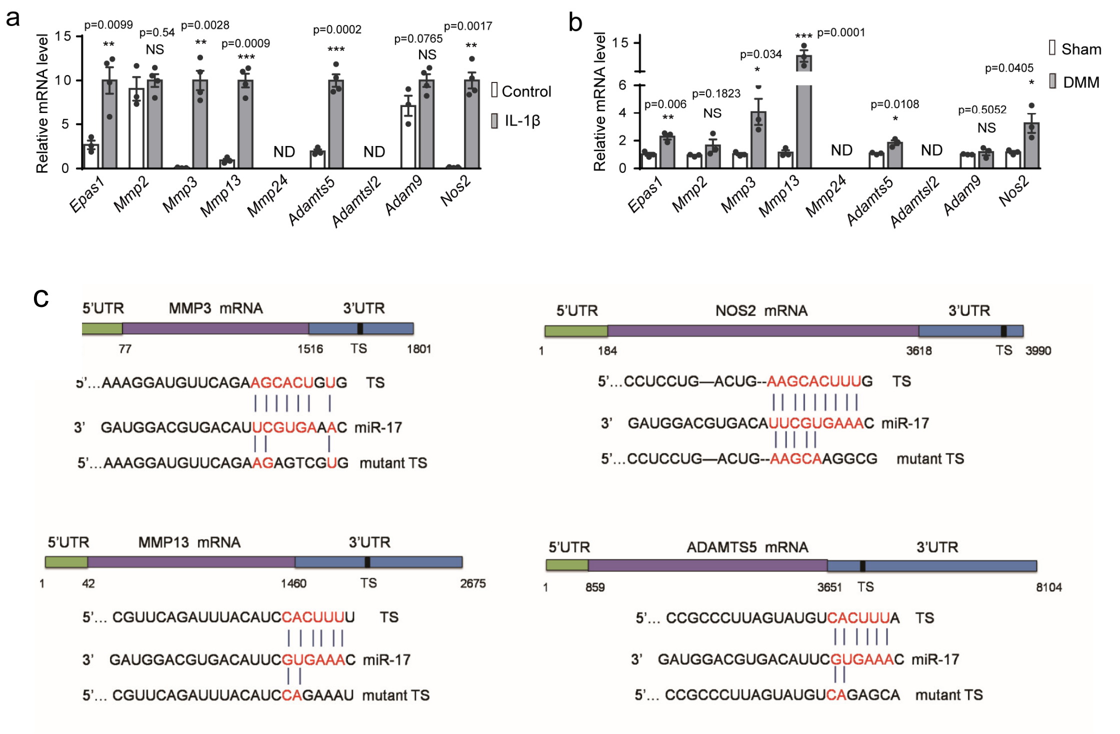

Supplementary Fig. 3 Identification of potential targets of miR-17 in OA chondrocytes.

a, Mouse articular chondrocytes were treated with or without IL-1β (5 ng/mL) for 24 h. The mRNA levels of several potential targets of miR-17, were determined by qRT-PCR. n = 3 biologically independent samples (control), n = 4 biologically independent samples (IL-1β).

b, qRT-PCR analysis of catabolic genes in knee cartilage of mice subjected to sham or DMM surgery for 4 weeks. n = 3 biologically independent samples per group.

c, MiR-17 binding sites and mutation strategies in the 3'-UTRs of *Mmp3, Mmp13, Nos2* and *Adamts5*.

Data are presented as mean ± s.e.m.. ND, no detection, NS, not significant, **P*< 0.05, ***P*< 0.01, and ****P*< 0.001 (two-sided Student's t-test).

Source data are provided as a Source Data file.

Supplementary Fig. 4

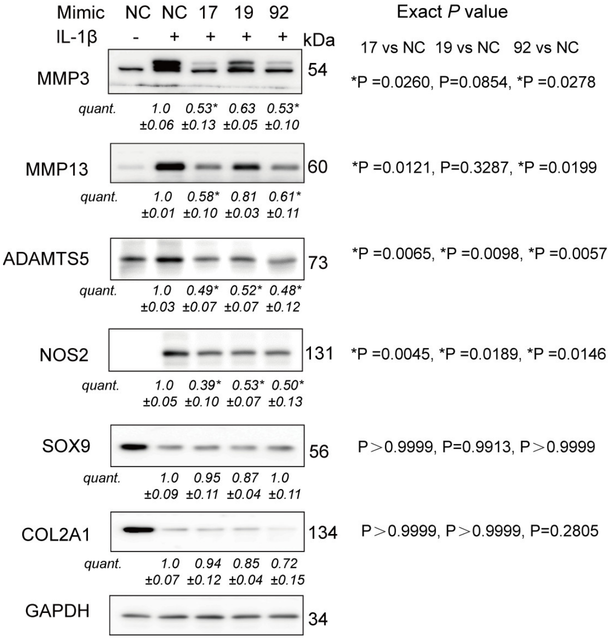

Supplementary Fig. 4 The effect of miR-17, miR-19 and miR-92 on catabolic and anabolic factors of IL-1β-treated chondrocytes.

Articular chondrocytes were transfected with miR-17, -19, -92 mimic (50 nM) or mimic NC and treated with or without IL-1β (5 ng/mL) for 24 h. The protein levels of catabolic and anabolic factors were determined by western blot followed by densitometry analysis. Blots are representative of three independent experiments. Data are presented as mean ± s.e.m.. *P< 0.05 versus IL-1β + mimic NC (one way ANOVA with Bonferroni's test). Source data are provided as a Source Data file.

Supplementary Fig. 5

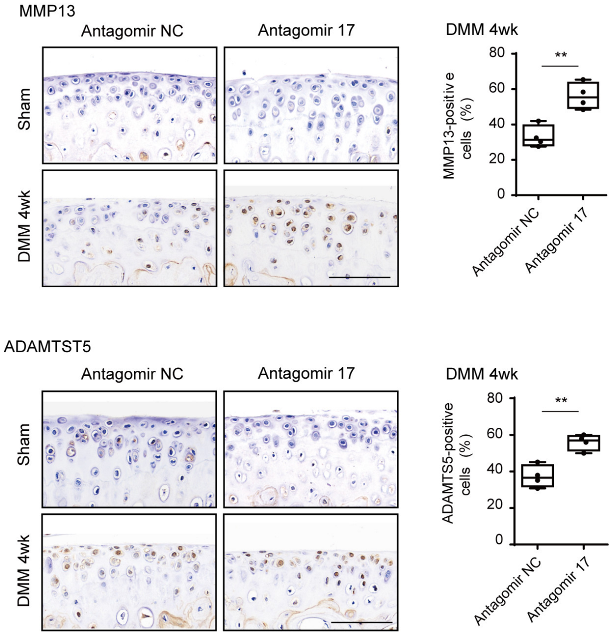

Supplementary Fig. 5 Inhibition of miR-17 increased expression of catabolic factors in DMM mice.

Representative images of IHC staining and quantification of MMP13^{+} and ADAMTS5^{+} cells in joint sections of mice subjected to sham or DMM surgery and intra-articular injections of antagomir-NC or antagomir-17. The injections were performed weekly from 1 week after surgery and the joints were collected at 4 weeks after surgery. n = 4 mice per group. Data are shown as box-plot. Box plots: center line, median; box limits, 25th to 75th percentiles; whiskers, min to max. $^{**}P< 0.01$ (Two-sided Student's t-test). All scale bars, 100 μm. Exact P value: 0.0031 (MMP13), 0.0023 (ADAMTS5).

Source data are provided as a Source Data file.

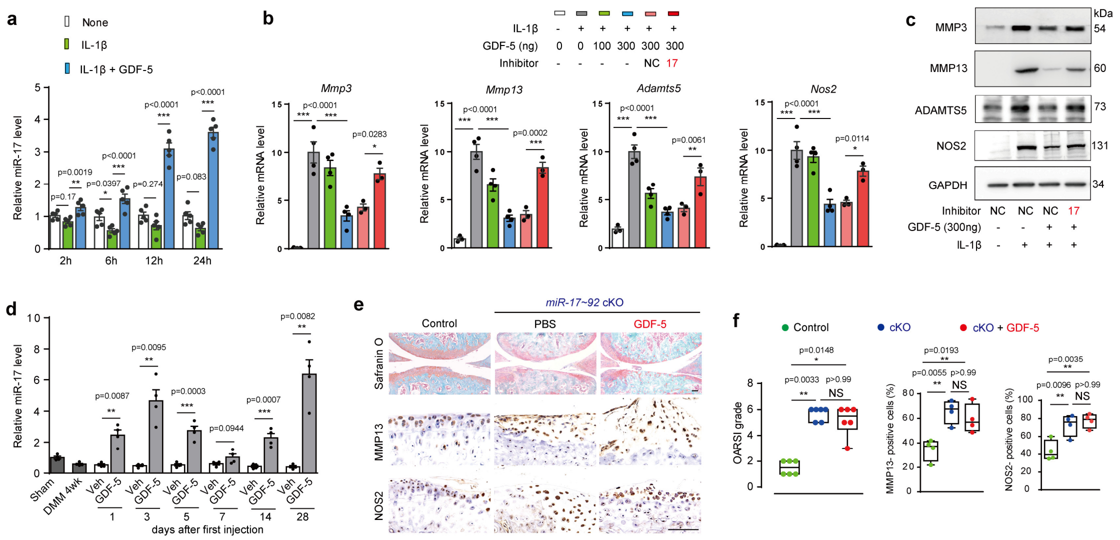

Supplementary Fig. 6 miR-17 was required for GDF-5 to suppress the expression of catabolic factors.

a, qRT-PCR detection of miR-17 expression induced by GDF-5 (300 ng/mL) in IL-1β-treated or un-treated chondrocytes at different time points. n = 5 biologically independent samples at each timepoint.

b, qRT-PCR analysis of catabolic factors in mouse primary articular chondrocytes treated with 5 ng/mL IL-1β and/or the indicated concentrations of GDF-5 for 24 h. The chondrocytes of last two groups were transfected with the miR-17 inhibitor (100 nM) or the inhibitor NC. n= 3 biologically independent samples (control, IL-1β+GDF-5+inhibitor NC, IL-1β+GDF-5+inhibitor 17); n=4 biologically independent samples (IL-1β, IL-1β+GDF-5 (100ng), IL-1β+GDF-5(300ng)).

c, Western blot analysis of MMP3, MMP13, ADAMTS5 and NOS2 in articular chondrocytes transfected with the miR-17 inhibitor (100 nM) or the control (inhibitor NC) and treated with IL-1β (5 ng/mL) alone or with a combination of IL-1β and GDF-5 (300 ng/mL) for 24 h. Blots represented three independent experiments.

d, qRT-PCR analysis of miR-17 in articular cartilage 0-28 days after intra-articular injections of GDF-5 (100 ng per injection). Weekly injections were performed for 4 weeks, beginning at 4 weeks after DMM operation. The Veh group was used as the control. n = 4 biologically independent samples per group at each timepoint.

e, f, Representative images of safranin O/fast green staining at 4 weeks after surgery, MMP13 and NOS2 immunostaining at 2.5 weeks after DMM surgery (e). OARSI grade,

and MMP13 and NOS2-positive cells (f) from knee joints of *miR-17~92* cKO mice and their controls were shown. PBS or GDF-5 (100 ng/injection) were injected weekly into knee joints of cKO mice beginning at 1 week after surgery. n= 6 mice per group (OARSI grade); n=4 mice per group (immunostaining). All scale bars, 100 μm.

The values shown are the mean ± s.e.m or box plot. Box plots: center line, median; box limits, 25th to 75th percentiles; whiskers, min to max. NS, not significantly different, **P*< 0.05 and ***P*< 0.01.****P*< 0.001. Two-sided Student's t-test for d; one way ANOVA with Bonferroni's test for a, b and positive cell counting in f; one way ANOVA (nonparametric) with Dunn's multiple comparisons test for OARSI grade in f. Source data are provided as a Source Data file.

Supplementary Fig. 7

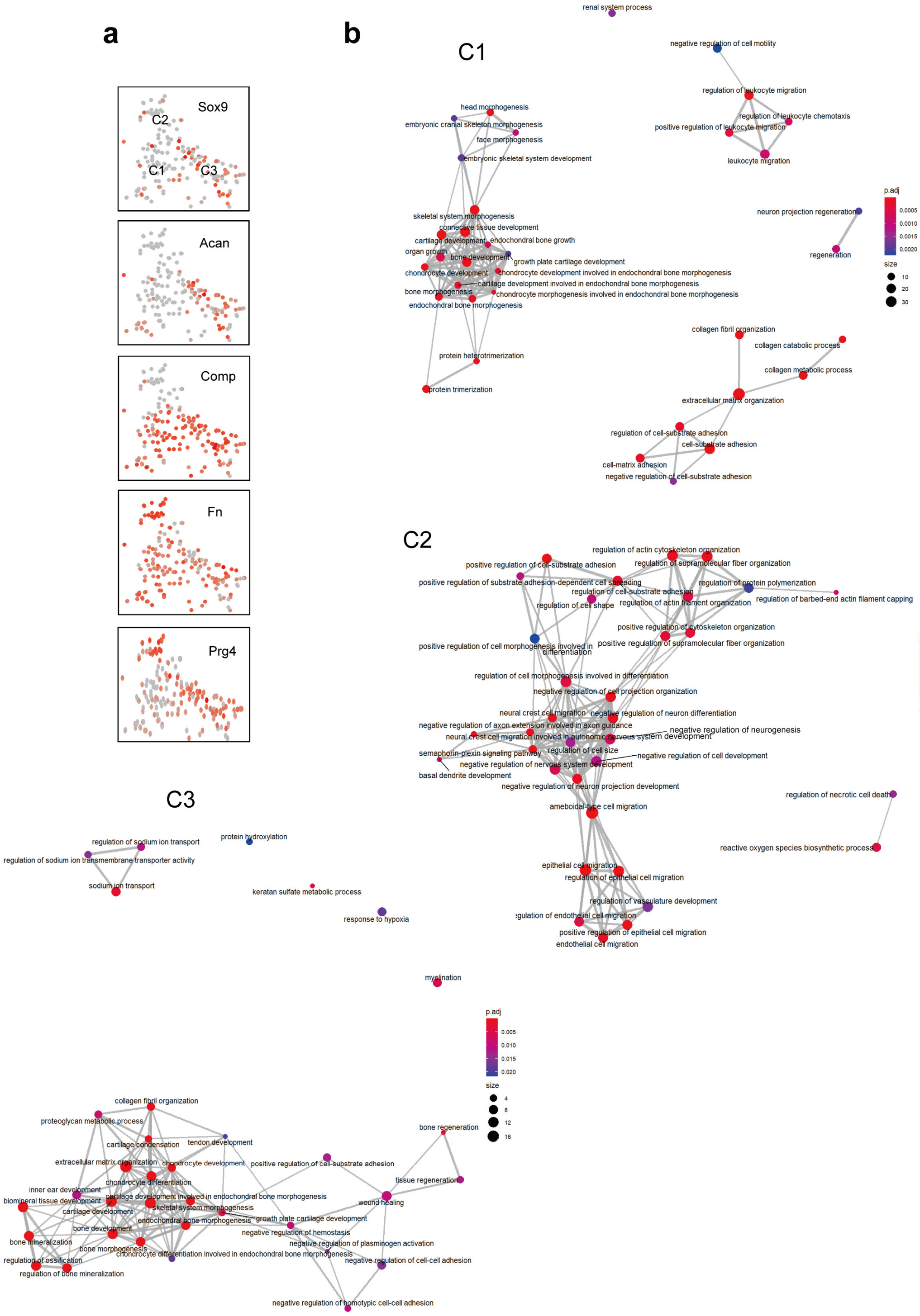

Supplementary Fig. 7 Identification of chondrocyte population from scRNA-seq and subdivision of three subsets.

a, Chondrocyte population (171 cells) and feature plot of chondrocyte marker genes.

b, GO analysis of C1-3 and the inter-relationship of these GO terms.

Supplementary Fig. 8

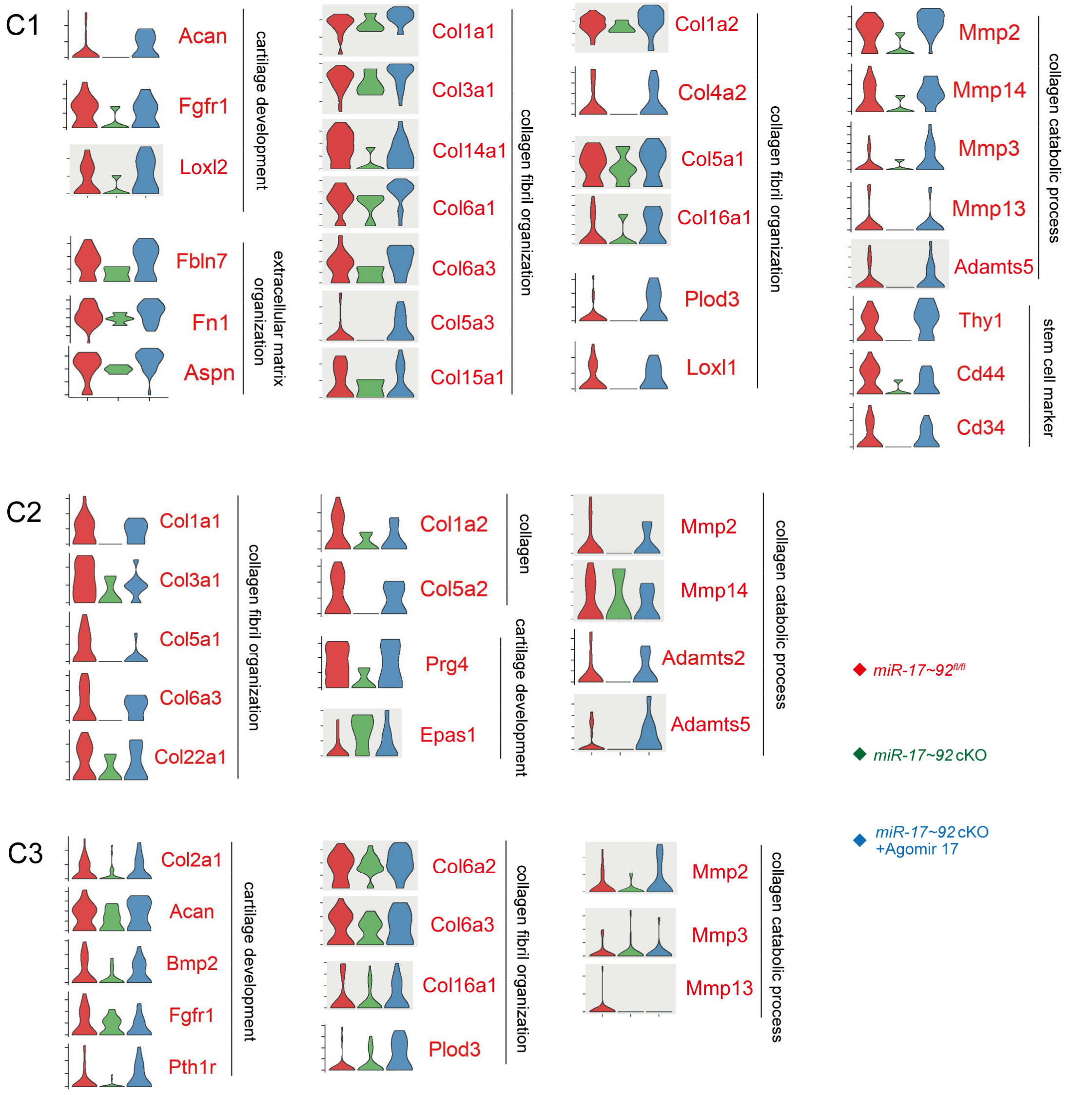

Supplementary Fig. 8 Changes of gene expression of anabolic and catabolic factors in *miR-17~92* cKO mice.

Violin plots showing changes in the levels of selected genes in clusters C1, C2, and C3.

Supplementary Fig. 9

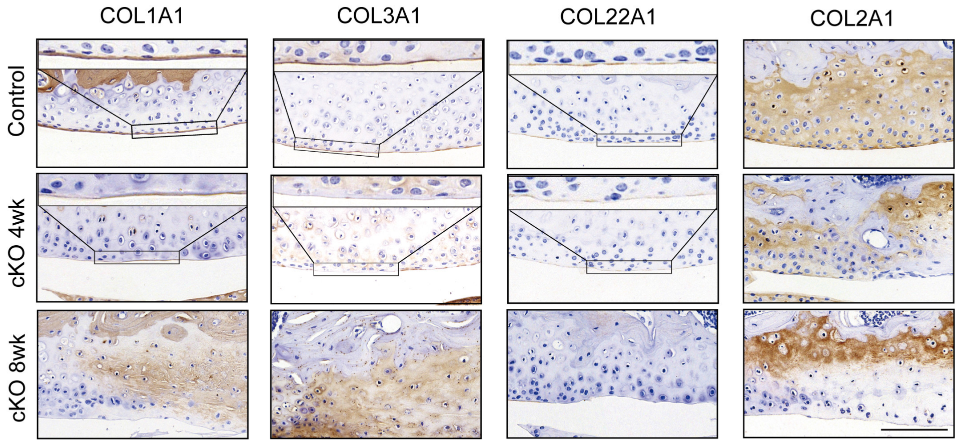

Supplementary Fig. 9 Changes of collagen expression in *miR-17~92* cKO mice. Immunohistology of collagen subtypes (COL1A1, COL3A1, COL22A1 and COL2A1) in femoral condylar cartilage from *miR-17~92* cKO mice at 4 and 8 weeks after injections of tamoxifen. *MiR-17~92*$^{fl/fl}$ mice were used as the control group. n= 3 mice per group. Scale bar, 100μm.

Supplementary Fig. 10

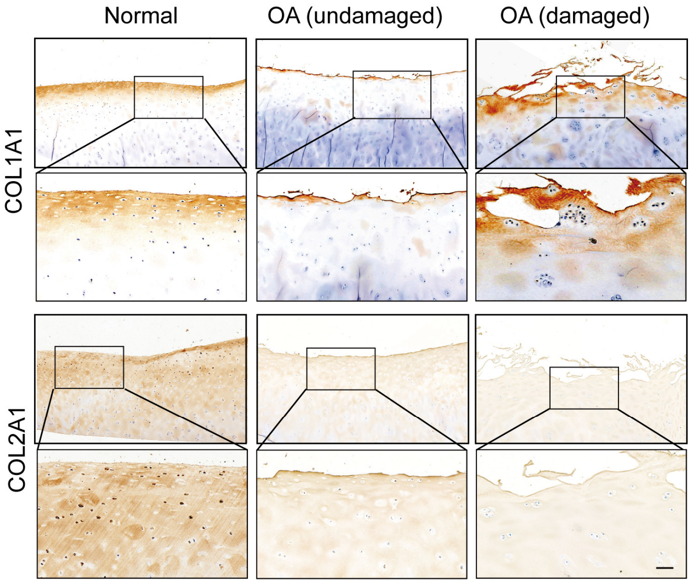

Supplementary Fig. 10 Changes of collagen expression in human OA samples Immunohistology of collagen subtypes (COL1A1 and COL2A1) in articular cartilage from normal individuals and OA patients (undamaged and damaged regions). n= 3 individual samples per group. Scale bar, 200μm.

Supplementary Table 1 Sequence of primers for mouse genes.

Gene	Primer (F=forward; R=reverse)	Amplicon size (bp)
Mmp3	F: 5'- TCCTGATGTTGGTGGCTTCAG -3'	102
	R: 5'- TGTCTTGGCAAATCCGGTGTA -3'	
Mmp13	F: 5'- ACTACCATCCTGCGACTCTTG -3'	111
	R: 5'- GTTTGCCAGTCACCTCTAAGC -3'	
Nos2	F: 5'- ACCTTGTTCAGCTACGCCTT -3'	112
	R: 5'- CATTCCCAAATGTGCTTGTC -3'	
Adamts5	F: 5'- GCCATTGTAATAACCCTGCACC -3'	292
	R: 5'- TCAGTCCCATCCGTAACCTTTG -3'	
Epas1	F: 5'- ATTGAGCGGGACTCTCGGAC -3'	213
	R: 5'- CGGGATTTCTCCTTCCTCAGC -3'	
Mmp2	F: 5'- CCAACTACGATGATGAC -3'	233
	R: 5'- ACCAGTGTCAGTATCAG -3'	
Mmp24	F: 5'- CTGGGCAGAACTGGTTAAA -3'	121
	R: 5'- ATCCCGTAAAACTGCTGCAT -3'	
Adamtsl2	F: 5'- GGGCAACAATCATCTTGGTTACT -3'	199
	R: 5'- CCGTCGGTACTTGACCACT -3'	
Adam9	F: 5'- CAGACTGCTGTGAGAGAAG -3'	252
	R: 5'- CATTCCTGCAGTTCCACCA -3'	
Lox	F: 5'- ACGCTGTGACATTCGCTACA -3'	115
	R: 5'- TGTCCAAACACCAGGTACGG -3'	
Plod2	F: 5'- TGTTTACCGAGTGTTTTGATGTTGT-3'	203
	R: 5'- GATGTACGGGGCATAGCCAA -3'	
P3h3	F: 5'- CAGTTGGGAGAGCCAAGACC -3'	131
	R: 5'- TCCGGGTCCTTGAAGCTAGT -3'	
Gapdh	F: 5'- GACTTCAACAGCAACTCCCAC -3'	125
	R: 5'- TCCACCACCCTGTTGCTGTA -3'	

Supplementary Table 2 Sequence of primers for site-specific mutants.

**Gene**	**Primer (F=forward; R=reverse)**
Mmp3	F: 5'-AAGGATGTTCAGAAGAGTCGTGTAGCTTACACTG-3'
	R: 5'-CAGTGTAAGCTACACGACTCTTCTGAACATCCTT-3'
Mmp13	F: 5'-TCAGATTTACATCCAGAAATATACAACCAATAAA-3'
	R: 5'-TTTATTGGTTGTATATTTCTGGATGTAAATCTGA-3'
Nos2	F: 5'-CTCCTGACTGAAGCAAGGCGGGTGACCACCAGGA-3'
	R: 5'-TCCTGGTGGTCACCCGCCTTGCTTCAGTCAGGAG-3'
Adamts5	F: 5'-GCCCTTAGTATGTCAGAGCATAAACTTGGTCCTA-3'
	R: 5'-TAGGACCAAGTTTATGCTCTGACATACTAAGGGC-3'
