# Supplementary material for: Dual functions of microRNA-17 in maintaining cartilage homeostasis and protection against osteoarthritis
Source: Nat Commun. 2022 May 4;13:2447. doi: 10.1038/s41467-022-30119-8 (PMC9068604; doi:10.1038/s41467-022-30119-8)
Supplement: Supplementary file 2 — Reporting Summary [file 41467_2022_30119_MOESM2_ESM.pdf]

nature portfolio

Corresponding author(s): Lei Cui

Last updated by author(s): Apr 13, 2022

Reporting Summary

Nature Portfolio wishes to improve the reproducibility of the work that we publish. This form provides structure for consistency and transparency in reporting. For further information on Nature Portfolio policies, see our Editorial Policies and the Editorial Policy Checklist.

Statistics

For all statistical analyses, confirm that the following items are present in the figure legend, table legend, main text, or Methods section.

n/a	Confirmed	
☐	☒	The exact sample size (*n*) for each experimental group/condition, given as a discrete number and unit of measurement
☐	☒	A statement on whether measurements were taken from distinct samples or whether the same sample was measured repeatedly
☐	☒	The statistical test(s) used AND whether they are one- or two-sided<br>*Only common tests should be described solely by name; describe more complex techniques in the Methods section.*
☒	☐	A description of all covariates tested
☒	☐	A description of any assumptions or corrections, such as tests of normality and adjustment for multiple comparisons
☐	☒	A full description of the statistical parameters including central tendency (e.g. means) or other basic estimates (e.g. regression coefficient) AND variation (e.g. standard deviation) or associated estimates of uncertainty (e.g. confidence intervals)
☐	☒	For null hypothesis testing, the test statistic (e.g. *F*, *t*, *r*) with confidence intervals, effect sizes, degrees of freedom and *P* value noted<br>*Give P values as exact values whenever suitable.*
☒	☐	For Bayesian analysis, information on the choice of priors and Markov chain Monte Carlo settings
☒	☐	For hierarchical and complex designs, identification of the appropriate level for tests and full reporting of outcomes
☒	☐	Estimates of effect sizes (e.g. Cohen's *d*, Pearson's *r*), indicating how they were calculated

Our web collection on statistics for biologists contains articles on many of the points above.

Software and code

Policy information about availability of computer code

Data collection	qRT-PCR: StepOne Real-Time PCR software v2.3.<br>For the histological and IHC analyses, images were scanned with a Pannoramic MIDI (3D HISTECH) and analyzed using CaseViewer 2.3 (3D HISTECH).<br>FISH images were captured with a laser-scanning confocal microscope (True Confocal Scanner SP5, Leica) using the LAS AF Lite 2.6.0 software (Leica).<br>Single cell RNA sequencing data was collected using the Chromium System and CellRanger platform (version 2.1.1, 10X Genomics).
Data analysis	Enrichment of GO terms in the “biological process” category was conducted using the Bioconductor GOstats package.<br>Cartilage thickness and western blot results were analyzed using Image J software version 1.8.0_112<br>ScRNA-seq analyses were performed with the R software package Seurat version 2.3.4.<br>graphs: GraphPad Prism v6.0.<br>Graphpad prism 6 was also used for statistical analysis as described in the Methods.

For manuscripts utilizing custom algorithms or software that are central to the research but not yet described in published literature, software must be made available to editors and reviewers. We strongly encourage code deposition in a community repository (e.g. GitHub). See the Nature Portfolio guidelines for submitting code & software for further information.

Data

Policy information about availability of data

All manuscripts must include a data availability statement. This statement should provide the following information, where applicable:

- Accession codes, unique identifiers, or web links for publicly available datasets
- A description of any restrictions on data availability
- For clinical datasets or third party data, please ensure that the statement adheres to our policy

Single-cell RNA-seq files have been uploaded to the websites, Accession: SRX10016273 (https://www.ncbi.nlm.nih.gov/sra/SRX10016273[accn]), SRX10016274 (https://www.ncbi.nlm.nih.gov/sra/SRX10016274[accn]), SRX10016275 (https://www.ncbi.nlm.nih.gov/sra/SRX10016275[accn]). Mouse reference, mm10 (Ensembl 84) can be found in https://support.10xgenomics.com/single-cell-gene-expression/software/release-notes/build#mm10_2.1.0. All the other data are available within the article and its supplementary information. Source data are provided with this paper. Uncropped blots are provided in source data.

Field-specific reporting

Please select the one below that is the best fit for your research. If you are not sure, read the appropriate sections before making your selection.

☒ Life sciences ☐ Behavioural & social sciences ☐ Ecological, evolutionary & environmental sciences

For a reference copy of the document with all sections, see nature.com/documents/nr-reporting-summary-flat.pdf

Life sciences study design

All studies must disclose on these points even when the disclosure is negative.

Sample size	Sample size for each experiment is indicated in the legend.<br>No statistical method was used to predetermine the sample sizes. The sample size for each experiment was determined based on previous studies (Jeon et al. 2017. Nat Med. PMID: 28436958; Zhen et al. 2013. Nat Med. PMID: 23685840; Huang et al. 2019. Nat Commun. PMID: 31253842) or our previous experiences.
Data exclusions	Samples obtained from animals were excluded from further analysis if there was evidence of joint infection or if the animal died after the procedure. For ScRNA-seq, genes expressed in <4 cells and cells expressing <200 genes were excluded. These exclusion criteria were preestablished.
Replication	For each figure panel, the number of independent experiments or biological replicates is indicated in the figure legends.<br>Western blot pictures are from a representative experiment and the number of independent repeats is clearly indicated in the figure legends.
Randomization	Wild type and knockout mice were randomly assigned into each experimental groups with various treatments. For in vitro experiments, cell cultures were randomly assigned to each experimental group and experiments were performed multiple times.
Blinding	For the mice surgeries, the operators were not blinded to the grouping information, because the operation procedures for sham and DMM surgery were different. For intraarticular injections, the operators were blinded to the grouping information. The investigators used sample ID and were not given grouping information during data collection. For histological analysis, the investigation were blinded to the group information. However, it would be easy to distinguished the histological manifestation between sham and DMM joints. Data analysis was confirmed by multiple investigators. Western blot, IHC and luciferase assay were frequently performed by participants other than the experiment designer.<br>We used unbiased computational approaches to analyze the single-cell data. Researchers processing all single-cell data were blinded to the study.

Reporting for specific materials, systems and methods

We require information from authors about some types of materials, experimental systems and methods used in many studies. Here, indicate whether each material, system or method listed is relevant to your study. If you are not sure if a list item applies to your research, read the appropriate section before selecting a response.

Materials & experimental systems

n/a	Involved in the study
☐	☒ Antibodies
☐	☒ Eukaryotic cell lines
☒	☐ Palaeontology and archaeology
☐	☒ Animals and other organisms
☐	☒ Human research participants
☒	☐ Clinical data
☒	☐ Dual use research of concern

Methods

n/a	Involved in the study
☒	☐ ChIP-seq
☒	☐ Flow cytometry
☒	☐ MRI-based neuroimaging

Antibodies

Antibodies used

IHC
anti-MMP13 (1:400, rabbit polyclonal, 18165-1-AP; Proteintech), anti-ADAMTS5 (1:200, rabbit polyclonal, DF13268; Affinity), anti-NOS2 (1:200, rabbit polyclonal, PA3-030A; Pierce), anti-COL1A1 (1:200, rabbit polyclonal, GB11022-1; Servicebio), anti-COL2A1 (1:200, rabbit polyclonal, GB11021; Servicebio), anti-COL3A1 (1:400, mouse monoclonal [FH-7A], ab6310; Abcam), and anti-COL22A1 (1:200, rabbit polyclonal, ab121846; Abcam).
IF
anti-MMP2 (1:500, rabbit polyclonal, GB11130; Servicebio), anti-CLIC5 (1:200, rabbit polyclonal, ab66630; Abcam), anti-APOD (1:300, rabbit polyclonal, DF7987; Affinity), anti-COL22A1 (1:200, rabbit polyclonal, ab121846; Abcam) and anti-CYTL1 (1:400, rabbit polyclonal, 15856-1-AP; Proteintech),
Goat anti-Rabbit IgG (H+L) Highly Cross-Adsorbed Secondary Antibody, Alexa Fluor 488 (1:300, A-11034; Invitrogen), Goat anti-Rabbit IgG (H+L) Cross-Adsorbed Secondary Antibody, Cyanine3 (1:400, A10520; Invitrogen).

WESTERN BLOT
anti-MMP3 (1:1000, rabbit monoclonal [EP1186Y], ab52915; Abcam), anti-MMP2 (1:1000, rabbit polyclonal, GB11130; Servicebio), anti-MMP13 (1:2000, rabbit polyclonal, 18165-1-AP; Proteintech), anti-ADAMTS5 (1:200, rabbit polyclonal, ab41037; Abcam), anti-NOS2 (1:1000, rabbit monoclonal [EPR16635], ab178945; Abcam), anti-SOX9 (1:1000, rabbit monoclonal [EPR14335-78], ab185966; Abcam), anti-COL2A1 (1:1000, rabbit polyclonal, 28459-1-AP; Proteintech), anti-HIF-1α (1:500, rabbit monoclonal [EPR16897], ab179483; Abcam), Lamin A/C (1:2000, mouse monoclonal [4C11], 4777S; Cell Signaling Technology), and GAPDH (1:1000, mouse monoclonal [2F40], T0004; Affinity),

Peroxidase AffiniPure Goat Anti-Mouse IgG (H+L), Horseradish Peroxidase (1:5000, 115-035-003; Jackson Immunoresearch), Peroxidase AffiniPure Goat Anti-Rabbit IgG (H+L), Horseradish Peroxidase (1:5000, 111-035-003; Jackson Immunoresearch).

Validation

MMP3
https://www.abcam.com/mmp3-antibody-ep1186y-ab52915.html
ADAMTS5
https://www.abcam.com/adamts5-antibody-ab41037.html
MMP13
http://www.ptgcn.com/Products/MMP13-Antibody-18165-1-AP.htm
NOS2
https://www.thermofisher.com/cn/zh/antibody/product/iNOS-Antibody-Polyclonal/PA3-030A
COL1A1
https://www.servicebio.com/html/Products/PrimaryAntibodies/Polyclonals/1325.html
COL2A1
https://www.servicebio.com/html/Products/PrimaryAntibodies/Polyclonals/1324.html
COL3A1
https://www.abcam.com/collagen-iii-antibody-fh-7a-ab6310.html
COL22A1
https://www.abcam.com/col22a1-antibody-ab121846.html
MMP2
https://www.servicebio.com/html/Products/PrimaryAntibodies/Polyclonals/1399.html
CLIC5
https://www.abcam.com/clic5-antibody-ab66630.html
APOD
http://www.affbiotech.cn/goods-11639-DF7987-APOD+Antibody.html
CYTL1
https://www.ptglab.com/Products/CYTL1-Antibody-15856-1-AP.htm
anti-MMP2 (1:1000, rabbit polyclonal, GB11130; Service),
https://www.servicebio.cn/goodsdetail?id=1399
anti-NOS2 (1:1000, rabbit monoclonal, ab178945; Abcam),
https://www.abcam.cn/inos-antibody-epr16635-ab178945.html
anti-SOX9 (1:1000, rabbit monoclonal, ab185966; Abcam),
https://www.abcam.cn/sox9-antibody-epr14335-78-ab185966.html

anti-COL2A1 (1:1000, rabbit polyclonal, 28459-1-AP; Proteintech),
https://www.ptgcn.com/products/Collagen-Type-II-Antibody-28459-1-AP.htm
anti-HIF-1α (1:500, rabbit monoclonal, ab179483; Abcam),
https://www.abcam.cn/hif-1-alpha-antibody-epr16897-ab179483.html
Lamin A/C (1:2000, mouse monoclonal, 4777S; Cell Signaling Technology),
https://www.cellsignal.cn/products/primary-antibodies/lamin-a-c-4c11-mouse-mab/4777?site-search-type=Products&N=4294956287&Ntt=4777s&fromPage=plp&_requestid=2925384
GAPDH (1:1000, mouse monoclonal, T0004; Affinity).
http://www.affbiotech.com/goods-6270-T0004-GAPDH+Antibody.html

Goat anti-Rabbit IgG (H+L) Highly Cross-Adsorbed Secondary Antibody, Alexa Fluor 488
https://www.thermofisher.com/cn/zh/antibody/product/Goat-anti-Rabbit-IgG-H-L-Highly-Cross-Adsorbed-Secondary-Antibody-Polyclonal/A-11034
Goat anti-Rabbit IgG (H+L) Cross-Adsorbed Secondary Antibody, Cyanine3
https://www.thermofisher.com/cn/zh/antibody/product/Goat-anti-Rabbit-IgG-H-L-Cross-Adsorbed-Secondary-Antibody-Polyclonal/A10520
Peroxidase AffiniPure Goat Anti-Mouse IgG (H+L), Horseradish Peroxidase (1:5000, 115-035-003; Jackson Immunoresearch),
https://www.jacksonimmuno.com/catalog/products/115-035-003
Peroxidase AffiniPure Goat Anti-Rabbit IgG (H+L), Horseradish Peroxidase (1:5000, 111-035-003; Jackson Immunoresearch).
https://www.jacksonimmuno.com/catalog/products/111-035-003

Eukaryotic cell lines

Policy information about cell lines

Cell line source(s)	HEK293T cells were purchased from ATCC (Cat No. CRL-11268) and cultured according to the culture methods of ATCC.
Authentication	Short tandem repeat profiling was used for authentication.
Mycoplasma contamination	Cell line tested negative for Mycoplasma Contamination.
Commonly misidentified lines (See ICLAC register)	No commonly misidentified cell lines were used in this study

Animals and other organisms

Policy information about studies involving animals; ARRIVE guidelines recommended for reporting animal research

Laboratory animals	All unique biological materials (genetically modified mouse strains) are available from standard commercial sources:<br>- C57BL/6J, male, at the age of 10 weeks old were used for surgery.<br>-12-month-old male WT C57BL/6 mice were used for assessment of aging-associated OA.<br>-For primary culture of mouse articular chondrocytes, the cells were isolated from the femoral condyles and tibial plateaus of 7-day old C57BL/6 mice.<br>-STOCK Mirc1tm1.1Tyj/J (miR-17~92fl) The Jackson Laboratory<br>#008458<br>- FVB-Tg(Col2a1-cre/ERT)KA3Smac/J The Jackson Laboratory<br>#006774<br>To generate Col2CreERT;miR-17~92fl/fl mice (miR-17~92 cKO), miR-17~92fl/fl mice were mated with Col2CreERT mice, and the resulting Col2CreERT;miR-17~92fl/+mice were then mated with miR-17~92fl/fl mice.<br>cKO mice were used at the age of 7 weeks, both male and female. For sham and DMM surgery, male mice were used.<br>Mice were housed under ambient temperature of 24±2°C, circulating air, constant humidity of 50±10% and a 12h:12h light/dark cycle.
Wild animals	The study did not use any wild animals.
Field-collected samples	This study does not include field-collected samples.
Ethics oversight	All animals were maintained in the Animal Facility of the Tongji University School of Medicine. The experimental procedures involving mice were performed in accordance with the Guidelines for the Care and Use of Laboratory Animals and approved by the Animal Care and Experiment Committee of Tongji University.

Note that full information on the approval of the study protocol must also be provided in the manuscript.

Human research participants

Policy information about studies involving human research participants

Population characteristics	Normal human articular cartilage was collected from the knee or hip joints of patients with osteosarcoma or trauma but without arthritis. Arthritic cartilage was sourced from individuals with OA. OA was diagnosed according to the criteria of the American College of Rheumatology. The patients without arthritis (1 male and 5 female) had an average age of 55.5 years

	(between 30 and 67 years of age). The patients with OA (4 male and 14 female) had an average age of 66.06 years (between 53 and 75 years of age).
Recruitment	All the cartilage tissue were from patients who underwent arthroplasty. Patients aged between 18 to 75 years old with osteosarcoma or trauma but without arthritis were recruited to provide healthy joint cartilage. The structural integrity of cartilage was confirmed by histological analysis. The participants ranged in age from 50 to 75 years with osteoarthritis were recruited, excluding disease such as diabetes, cancers, rheumatic arthritis and other bone disease. Sex, weight and height of patients were not controlled. Participants were recruited by participating surgeons (Kaijin Guo). There was no self-selection bias in this study.<br>All patients were recruited at the Department of Orthopedics, Affiliated Hospital of Xuzhou Medical University.<br>All patients signed an informed consent.
Ethics oversight	The collection of discarded human tissues was approved by the Research Ethics Board of Affiliated Hospital of Xuzhou Medical University. The patients did not receive participant compensation. The study design and conduct complied with all relevant regulations regarding the use of human study participants and was conducted in accordance with the criteria set by the Declaration of Helsinki.

Note that full information on the approval of the study protocol must also be provided in the manuscript.
